# Demographics and Length of Stay for Osteomyelitis in Opioid Drug Users: A Unique Population with High Healthcare Costs

**DOI:** 10.7759/cureus.4339

**Published:** 2019-03-28

**Authors:** Eric Yuschak, Stacy Chase, Furqan Haq, Christian Vandever

**Affiliations:** 1 Family Medicine, St. Petersburg General Hospital, St. Petersburg, USA; 2 Internal Medicine, Oak Hill Hospital, Tampa, USA; 3 Statistics, Hospital Corporation of America, Nashville, USA

**Keywords:** osteomyelitis, diabetes, opioids

## Abstract

Osteomyelitis is an infection of the bone. Risk factors include, but are not limited to, diabetes and intravenous drug use. The hypotheses for the primary objectives of this study were that the opioid epidemic would cause a younger population of patients to be seen with osteomyelitis and the treatment for this population has special considerations including longer hospitalization for proper intravenous antibiotics. This retrospective chart review compared 2,150 cases of osteomyelitis in the Hospital Corporation of America (HCA Healthcare) West Florida Division. A sample group of osteomyelitis with diabetes was compared to a group with reported opioid use. The results showed a significantly younger age at which the osteomyelitis was occurring in opioid drug users and a significantly longer hospitalization for the treatment. With the rising costs of healthcare and the continuing growth of drug abuse, the 11.501-year younger age difference (95% confidence interval [9.204, 13.799], *p-*value <0.001) and 4.992-day longer hospitalization (95% confidence interval [3.053, 6.931], *p*-value <.001) can raise awareness on an additional impact of drug abuse on healthcare costs.

## Introduction

Osteomyelitis is an infection involving the bone. A study by Kremers showed the incidence to be 21.8 per 100,000 person-years during 1969-2009 [1]. There are multiple risk factors, but diabetes and intravenous (IV) drug use are two of the most common with different routes of infection. Signs and symptoms can include pain, swelling, warmth, and erythema of the involved area as well as systemic signs of infection such as fever defined as the temperature >38°C. The workup for suspected osteomyelitis includes a thorough history and physical, lab evaluation including white blood cell count, C-reactive protein (CRP), erythrocyte sedimentation rate (ESR), blood cultures, and radiographic imaging [2]. CRP is also used to monitor the effectiveness of treatment. The diagnosis and causative agent can be established by culturing the involved bone via biopsy. Obtaining an accurate culture helps establish susceptibility data for antibiotic treatment.

Osteomyelitis can occur both hematogenously and non-hematogenously. Hematogenous osteomyelitis refers to spread from an organism in the setting of bacteremia, while non-hematogenous refers to spread from the adjacent soft tissue or direct inoculation [2]. IV drug use typically results from hematogenous spread with *Staphylococcus*, being the most common organism, and vertebral body involvement, being the most common location. Diabetes typically causes non-hematogenous osteomyelitis secondary to *Staphylococcus aureus* from diabetic foot wounds and decubitus ulcers. Signs and symptoms can be masked in diabetics from peripheral neuropathy. Therefore, these symptoms are typically seen in diabetic patients with poorly controlled and/or prolonged disease.

Osteomyelitis treatment varies, and antibiotic selection and duration of therapy are commonly debated. Optimal duration is uncertain, but a minimum of four to six weeks is typical and empiric antibiotic coverage should cover against methicillin-resistant *Staph. aureus* and aerobic Gram-negative bacilli. Prolonged hospital stays to complete the course of treatment are a concern for patients, providers, and hospital systems. The increased utilization of intravenous catheters to complete treatment as an outpatient has made hospitalizations shorter; however, in patients with a history of IV drug use, this may allow them direct venous access for potential illicit drug use. Although guidelines are currently not available, in an article featured in *The Hospitalist*, Conrad mentions, “One of the guiding principles should be, despite financial pressures that the primary focus is on appropriate care of this vulnerable population” [3]. This is supportive for keeping the patient hospitalized to remove the temptation despite the cost. In comparison, for patients without IV drug abuse history, including non-hematogenous osteomyelitis secondary to diabetes, antibiotics can be arranged to complete as outpatient with either return to infusion centers or home health assistance. With our study results, this problem could be addressed by supporting programs to help those affected by drug addiction.

Drug addiction in the United States is a growing problem. Death rates from overdoses continue to climb as seen in the report by the National Institute on Drug Abuse [4]. In addition, special considerations have to be made regarding the appropriate treatment of comorbidities. Comorbidities such as skin infections, abscesses, endocarditis, and osteomyelitis have increased prevalence in patients who abuse IV drugs. The British Journal of Dermatology reported that in confiscated injected drugs, 89% were contaminated with disease-causing pathogens in addition to those pathogens from dirty injection sites and non-sterile equipment [5]. The community of St. Petersburg and Tampa Bay is located within Hospital Corporation of America's (HCA Healthcare) West Florida Hospital Division which is the population of this study. The National Survey on Drug Use and Health collected data showed that the drug use prevalence is similar in this population to the national prevalence which helps predict the validity of this retrospective study [6].

Three articles were related to this study’s interest as they help diagnosis protocol and antibiotic selection for treatment. First, a retrospective study by Colip looked at imaging the spine with magnetic resonance imaging (MRI) in the workup of intravenous drug use (IVDU) with acute low back pain. Given the most common site of involvement for hematogenous osteomyelitis in IVDU is the vertebra, they found the imaging is justified despite pressure on MRI scanner, tech, and interpretation [7]. This is significant as it helps diagnosis of the condition earlier which can help contain the infection requiring less intense therapy that could relate to the length of hospitalization, a variable this study is interested in. The second study was related to the first as it was a case study by Ali featuring a 50-year-old patient with a history of IVDU and chronic back pain [8]. The take-home from this study was once again early imaging and high clinical suspicion are necessary to correctly diagnose this condition which was missed on multiple previous emergency room visits. Finally, the third article by Nather discussed osteomyelitis in the diabetic foot. In this article, they identified the distribution of antibiotic resistance and that a wound area exceeding 4 cm is at increased risk of osteomyelitis [9]. This is significant to this study as diabetic osteomyelitis tends to present differently than osteomyelitis in IVDU.

## Materials and methods

This study is a retrospective chart review using HCA Healthcare’s data bank. Institutional review board (IRB) exemption was obtained. The HCA Healthcare West Florida division was isolated, and the International Classification of Disease (ICD) 10 codes were used to gather relevant data from the two-year period of 2016-2018. Inclusion criteria included age 18-70 years with the diagnosis of osteomyelitis and diabetes or opioid use. These two groups were chosen for comparison because diabetes is a common comorbidity of the disease, while drug use is less common comorbidity and both can be considered modifiable with interventions. The data were de-identified and secured on password-protected computers and programs with limited access to the principal investigators and HCA Healthcare data team and statisticians. The subpopulations were then analyzed to determine the average ages and the length of stays. An independent sample *t-*test with equal variances not assumed was used to compare ages, while a linear regression model was used to compare the length of stays in order to answer the primary objectives of whether the opioid subgroup was a younger population experiencing osteomyelitis and whether the opioid subgroup required a longer length of stay for treatment.

## Results

A total of 2,150 patients were identified for meeting the inclusion criteria. The data were divided into the subgroups of diabetics and opioid drug users for comparison. The diabetic group contained 2,038 patients. The opioid drug group contained 112 patients. The diabetic group averaged an age of 56.65 years with a standard deviation of 9.433 years and averaged a length of stay of 9.59 days with a standard deviation of 9.389 days. The opioid group averaged an age of 45.15 years with standard deviation of 12.079 years and averaged length of stay of 14.49 days with a standard deviation of 16.877. The independent samples *t*-test showed these data were significant for an average age 11.501 years younger in the opioid group (95% confidence interval [9.204, 13.799], *p*-value <0.001). The regression for the length of stay showed a significantly longer stay for the opioid group with the length of stay 4.992 days longer (95% confidence interval [3.053, 6.931], *p-*value <0.001; Table 1).

**Table 1:** Group statistics

	Opioid Use	Patients (N)	Mean	Standard Deviation	Standard Error of Mean
Length of Stay (Days)	0 (Diabetes)	2038	9.59	9.389	0.208
	1 (Opioid)	112	14.49	16.877	1.595
Age (Years)	0 (Diabetes)	2038	56.65	9.433	0.209
	1 (Opioid)	112	45.15	12.079	1.141

## Discussion

The strengths of this study include comprehensive data from HCA Healthcare that was from a large database and analyzed with professional research assistance. This study has growth potential by including more divisions of HCA Healthcare, as similar data are available for greater validity. Further analysis of years from previous decades to observe if the percentages of opioid-associated osteomyelitis are increasing would be another potential way to expand this study, however; that data was not available in the data bank. Finally, this study could be expanded to look at the treatment received by the groups as hypotheses were made as the causes for variations in length of stay.

There were several limitations to the research project. Being a retrospective chart review, improper or missed documentation may limit the ability to collate data. Opiate use may be underrepresented in the population observed due to intentional or unintentional misrepresentation of opiate use history by patients. Opioid use is self-reported and in the social history of documentation. With this in mind, of our 2,150 cases of osteomyelitis, only 5.2% were reported and documented opioid use. It was also hard to compare the location of the osteomyelitis being treated which could be amenable to certain treatment protocols. With these limitations in mind, the group for osteomyelitis with opioid use was much smaller, and the results above were obtained.

## Conclusions

This retrospective chart review significantly showed that patients with osteomyelitis and opioid use were, on average, younger in age and required a longer hospitalization than those with osteomyelitis and diabetes. As previously discussed, osteomyelitis is a chronic comorbidity of the diabetes and correlates to the older age of that population of patients. These patients are able to have catheters placed to allow for discharge from the hospital and outpatient management with antibiotics for the necessary time period. Related comorbidities found in longstanding diabetic patients include compromised circulation in the extremities which may necessitate a procedure such as an amputation that also decreases the length of hospitalization. The studied osteomyelitis cohort who used IV drugs was younger than the diabetic osteomyelitis group. The lack of central catheter line availability for outpatient management in this cohort leads to prolonged hospitalization. For most medical problems, younger patients require less treatment in a hospital, but this is a special population. And because they are generally younger with healthy limbs, procedures such as amputations are avoided if at all possible. Although this rational is a hypothesis, it would account for the results seen. This leaves prolonged hospitalizations and close monitoring as the best treatment option which costs more healthcare dollars for the younger population of patients. 
